# Functional outcomes and quality of life after AcrySof IQ Vivity intraocular lens implantation in a real-world study

**DOI:** 10.1038/s41598-024-69960-w

**Published:** 2024-09-04

**Authors:** Federico Giannuzzi, Matteo Mario Carlà, Fabio Margollicci, Gaetano Di Stefano, Andrea Molle, Lorenzo Hu, Francesco Boselli, Monica Maria Pagliara, Domenico Lepore, Fernando Molle, Stanislao Rizzo

**Affiliations:** 1 Ophthalmology Unit, Agostino Gemelli University Polyclinic, Rome, Italy; 2 Catholic University “Sacro Cuore”, Rome, Italy; 3 Ocular Oncology Unit, Fondazione Policlinico Universitario A. Gemelli, IRCCS”, Rome, Italy; 4 Consiglio Nazionale delle Ricerche, Istituto di Neuroscienze, Pisa, Italy; 5 Ophthalmology Department, “Fondazione Policlinico Universitario A. Gemelli, IRCCS”, Largo A. Gemelli, 8, Rome, Italy

**Keywords:** Applied optics, Eye diseases

## Abstract

The extended depth-of-focus AcrySof IQ Vivity intraocular lens technology offers promising features for presbyopia management, evaluated in this research in a 6 months real-world setting. Prospective interventional mono-centric study including 40 patients who underwent elective bilateral phacoemulsification. We performed one pre-operative visit (V0) and one evaluation six months post-operatively (V1), evaluating uncorrected and corrected visual acuity for near (UNVA/CNVA), intermediate (UIVA/CIVA) and far (UDVA/UCVA), slit-lamp evaluation, tomography with static pupillometry, endothelial cell count and contrast sensitivity chart. In order to assess post-operative Quality of Life, we administered the patients McAlinden’s Quality of Vision test and Morlock’s Patient-Reported Spectacle Independence Questionnaire. We divided eyes in with Toric-IOL and with non-Toric IOL. A total of 36 eyes received non-tonic IOL implantation, whereas 44 eyes received toric IOL implantation. There were no statistically significant disparities observed in visual outcome measures and contrast sensitivity between the toric group and the non-toric group. Furthermore, we assessed the predictive preoperative refractive astigmatism (PPRA) and residual refractive astigmatism (RRA) in both cohorts, and no statistical significance was found between the two cohorts (*p* = 0.08). Twenty-one (53%) patients reported total independence from their glasses at all distances. The mean difference between the predicted and measured refractive error, as calculated by spherical equivalent, was 0.09 D. AcrySof IQ Vivity is a well-tolerated and effective IOL with optimal refractive target for both distant and intermediate vision, needing slight spherical addition for the best near vision. Great questionnaire-based satisfaction was reported by the patients.

## Introduction

Cataracts affect millions globally, with over half of blindness cases attributed to them. The condition, prevalent among older adults, significantly impacts quality of life beyond age 70^1,2^. Cataract surgery is the leading surgical procedure in developed nations^3^. In Italy, a survey found two-thirds of those 70 and older had advanced cataracts, with over half experiencing significant vision decline^4^.

As surgical techniques and technologies continue to advance, cataract surgery has transitioned from a rehabilitative intervention to an actual refractive procedure. The incorporation of intraocular lenses (IOL) in the treatment of presbyopia following cataract surgery has become commonplace in high-quality cataract procedures, as there is a growing demand for reduced spectacles dependence after surgery. In recent decades, there has been remarkable progress in IOL technology, providing ophthalmologists with a broader selection of options for addressing presbyopia through the implantation of “premium” IOLs.

The implantation of multifocal IOLs for correcting presbyopia was first performed in human eyes in 1986. Despite being the pioneering type of IOL, their widespread adoption was initially gradual^5,6^. Both Cochrane reviews and meta-analysis studies have demonstrated that multifocal IOLs offer higher rates of spectacle independence compared to monovision^7^. However, there was no substantial difference observed between multifocal IOLs and monovision in terms of uncorrected distance visual acuity (UDVA), uncorrected intermediate visual acuity (UIVA), and uncorrected near visual acuity (UNVA)^7^. A recent Cochrane review suggested that monovision resulted in fewer symptomatic higher-order aberrations compared to multifocal IOLs, although the estimate uncertainty was high^8^. Additionally, meta-analysis studies indicated that patients who received multifocal IOLs experienced more frequent and bothersome subjective visual disturbances, such as glare and haloes, in comparison to those who received monovision^7^.

The recently introduced Extended Depth of Focus (EDOF) lenses differ significantly from multifocal IOLs. EDOF lenses are specifically designed to create a continuously elongated focal point along the visual axis, unlike the biphasic or triphasic peaks typically seen in bifocal or trifocal multifocal IOLs. This phenomenon is achieved by utilizing different optical designs, such as multifocal or pinhole optical configurations, and by harnessing spherical aberration and chromatic aberration^9–11^. From a clinical perspective, patients who undergo EDOF lens implantation often require a spherical addition to achieve optimal near vision. However, these lenses have as a premise to lessen spectacle dependance for intermediate vision^12–14^.

The new AcrySof IQ Vivity IOL (Alcon Laboratories Inc, Fort Worth, TX, USA) is the first IOL that addresses presbyopia using non-diffractive EDOF optics. The IOL is made of an hydrophobic acrylate/methacrylate copolymer with UV and blue light filters, has a refractive index of 1.55 at 35 °C, an optic diameter of 6 mm and an overall length of 13 mm. The IOL spherical power ranges from + 10.0 to + 30.0 D and astigmatism correction up to 3.75 (T0–T6) is currently available^15^.

The AcrySof IQ Vivity IOL features two smooth surface transition elements located in the central 2.2 mm region of the IOL^2^. The first transition element is a slightly elevated smooth plateau measuring approximately 1 μm in height, while the second transition element involves a subtle curvature change.

The AcrySof IQ Vivity IOL provides patients with distance, intermediate, and functional near vision capabilities while offering the visual disturbance profile of a monofocal IOL^16^.

The primary outcome of the present study is to assess the safety, the improvements in visual acuity and quality of life and the rate of spectacle independence at 6 months following the binocular implantation of the AcrySof IQ Vivity IOL. Secondary outcomes included the assessment of postoperative refractive error including residual astigmatism, rate of postoperative toric IOL rotation and contrast sensitivity.

## Results

We enrolled 40 patients, 24 (60%) male and 16 (40%) female. Mean age at evaluation was 68.5 ± 6.3 years (range 57–82 years). Mean follow up was 6.3 ± 0.8 months. Table 1 shows baseline characteristics of the cohort.

**Table 1:** Demographics characteristics of the study cohort.

Parameter	Value
Eyes (n)	80
Patients (n)	40
Age (y)	68.5 ± 6.3
Range	57–82
Male sex (%)	24 (60%)
CDVA (logMAR)	0.34 ± 0.18
UDVA (logMAR)	0.48 ± 0.24
Mean contrast sensitivity (%)	0.05 ± 0.02
Photopic pupil size between 3–4 mm (%)	83
Mean axial length (mm) ± SD	24.69 ± 1.50
Mean endothelial cell count (cells/mm^2^)	2586 ± 329
Mean predicted refractive error (D)	− 0.25 ± 0.18
Mean IOL power (D) ± SD	21.25 ± 4.59
Toric IOL implantation (%)	44 (55%)

Data are expressed in mean ± SD.

A total of 36 eyes received non-tonic IOL implantation, whereas 44 received toric IOL implantation.

Table 2 summarizes visual outcomes of the two cohorts.

**Table 2:** Distance, intermediate, and near visual acuities 6 months post-operatively. Significant values are in italics.

Parameter	Entire cohort	Non-toric cohort	Toric cohort	
	Visual acuity (LogMAR)	Visual acuity (LogMAR)	Visual acuity (LogMAR)	p-value
Distance (6 m)				
UDVA	0.04 ± 0.06	0.04 ± 0.05	0.04 ± 0.06	*0.13*
Binocular UDVA	0.02 ± 0.03	0.02 ± 0.03	0.02 ± 0.03	
CDVA	− 0.04 ± 0.06	− 0.04 ± 0.07	− 0.03 ± 0.07	*0.18*
Binocular CDVA	− 0.05 ± 0.07	− 0.05 ± 0.07	− 0.05 ± 0.07	
Intermediate (80 cm)				
UIVA	0.04 ± 0.05	0.02 ± 0.05	0.05 ± 0.06	*0.08*
Binocular UIVA	− 0.03 ± 0.06	0.02 ± 0.03	0.02 ± 0.03	
Binocular CIVA	− 0.03 ± 0.06	− 0.03 ± 0.06	− 0.03 ± 0.06	
Intermediate (60 cm)				
UIVA	0.06 ± 0.07	0.04 ± 0.05	0.08 ± 0.08	*0.06*
Binocular UIVA	0.04 ± 0.07	0.04 ± 0.07	0.04 ± 0.07	
Binocular CIVA	− 0.03 ± 0.06	− 0.03 ± 0.06	− 0.03 ± 0.06	
Near (40 cm)				
UNVA	0.17 ± 0.11	0.16 ± 0.06	0.18 ± 0.15	*0.07*
Binocular UNVA	0.11 ± 0.04	0.11 ± 0.04	0.11 ± 0.04	
Binocular CNVA	− 0.04 ± 0.06	− 0.04 ± 0.06	− 0.04 ± 0.06	

Data are expressed in mean ± SD.

*CDVA* corrected distance visual acuity, *CIVA* corrected intermediate visual acuity, *UDVA* uncorrected distance visual acuity, *UIVA* uncorrected intermediate visual acuity, *UNVA* uncorrected near visual acuity.

Furthermore, we assessed the predictive preoperative refractive astigmatism (PPRA) and residual refractive astigmatism (RRA) in both cohorts, and no statistical significance was found between the two cohorts (*p* = 0.08). During follow-up in either cohorts, no adverse events were observed, including secondary surgical interventions, subjective posterior capsular opacification (PCO) assessment, posterior capsulotomy, and increased intraocular pressure.

### Visual outcomes

**Figure 1:**
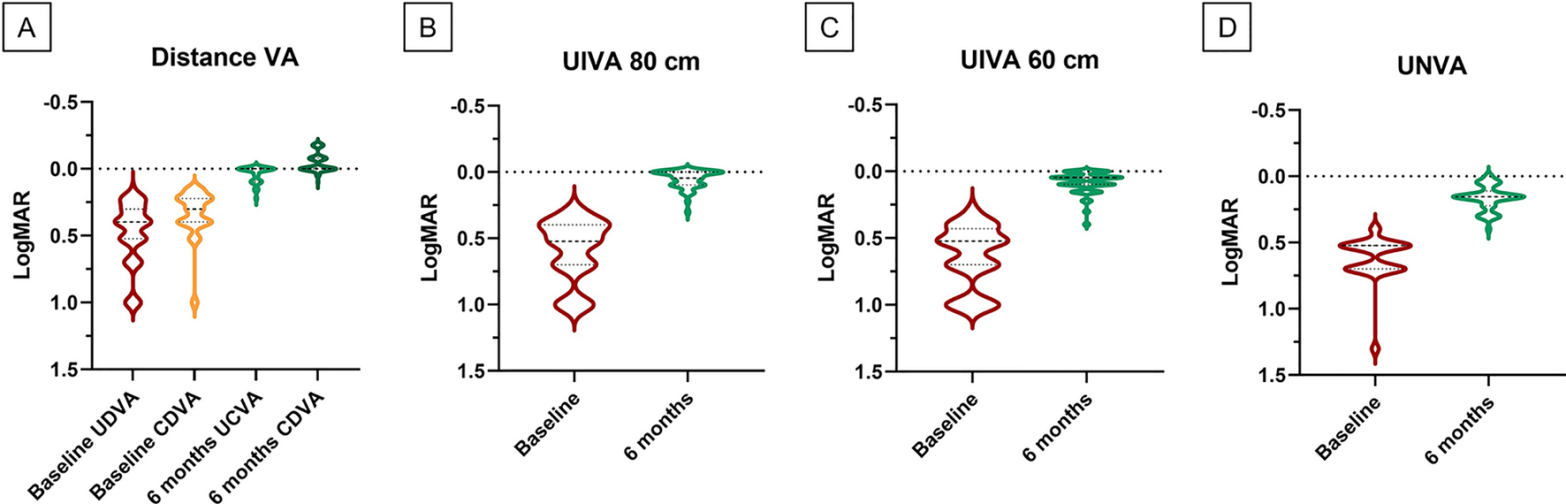
Violin plots showing the comparison, from left to right, between (**A**) Uncorrected and Corrected Distance Visual Acuity (Baseline and 6-month follow up), (**B** and **C**) Uncorrected Intermediate Visual Acuity at 80 cm and 60 cm (Baseline and 6-month follow-up), (**D**) Uncorrected Near Visual Acuity (Baseline and 6-month follow-up) in monocular assessment.

Visual improvements of the entire cohort are visible in Fig. 1.

### Non-toric IOL cohort

Baseline mean UDVA and CDVA were 0.42 ± 0.23 LogMAR and 0.35 ± 0.19 LogMAR, respectively. Mean UIVA at 80 cm and 60 cm were 0.53 ± 0.23 LogMar and 0.62 ± 0.25, respectively. Mean UNVA was 0.62 ± 0.18 LogMar. Mean contrast sensitivity was 2.2 ± 0.05%.

At V1, monocular mean UDVA was significantly improved to 0.04 ± 0.05 LogMar (*p* < 0.01), while average monocular CDVA was − 0.04 ± 0.07 LogMar (*p* < 0.01). For intermediate vision, UIVA at 80 cm and 60 cm was 0.02 ± 0.05 LogMar (*p* < 0.01) and 0.04 ± 0.05 LogMar (*p* < 0.01), respectively. At 40 cm, UNVA were 0.16 ± 0.06 LogMar (*p* < 0.01).

In the non-toric IOL group, postoperative residual refractive astigmatism of 0.75 D or less was seen in 35 out of 36 eyes (97%), with 1 eye (3%) showing a residual astigmatism of 1.00 D. At V1, mean contrast sensitivity was 1.6 ± 0.03%, significantly improved from baseline (*p* = 0.03). A summary of visual outcomes is visible in Table 2

### Toric IOL cohort

At V0, mean UDVA was 0.51 ± 0.23 LogMar, while mean CDVA was 0.38 ± 0.16 LogMar. Mean UIVA at 80 cm and 60 cm were 0.58 ± 0.23 LogMar and 0.63 ± 0.22, respectively. Finally, mean UNVA was 0.68 ± 0.20 LogMar.

At V1, monocular mean UDVA improved to 0.04 ± 0.06 LogMar (*p* < 0.01), while monocular average CDVA reached 0.03 ± 0.07 LogMar (*p* < 0.01). For intermediate vision, UIVA at 80 cm and 60 cm were 0.05 ± 0.06 LogMar and 0.08 ± 0.08 LogMar, respectively, both improved from baseline values (*p* < 0.01). At 40 cm, UNVA was 0.18 ± 0.15 LogMar (*p* < 0.01). Finally, mean contrast sensitivity changed from 2.5 ± 0.7% at V0 to 1.9 ± 0.4% at V1 (*p* = 0.02).

**Figure 2:**
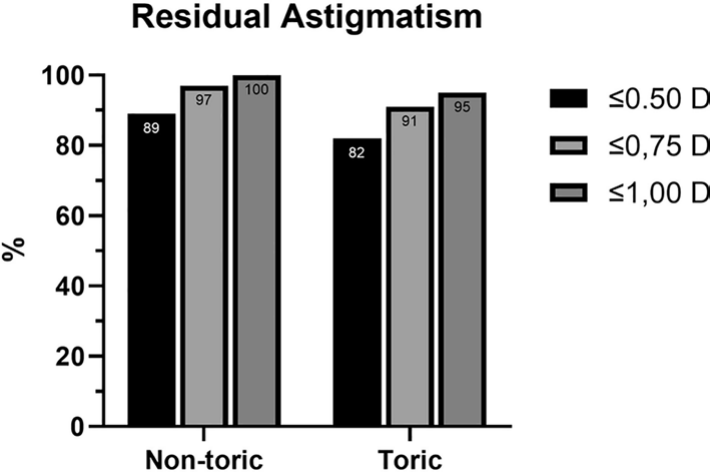
Histograms showing the percentage of residual astigmatism in non-toric and toric AcrySof IQ Vivity IOL implantation.

In this cohort, 36 out of 44 eyes (82%) showed a RRA of 0.50 D or less, while 42 out of 44 eyes (95%) had a residual astigmatism of 1.00 D or less. As of the remaining 2 out of 44 eyes (5%), residual astigmatism was 1.25 D one eye, while in the other was 2.00 D (with a postoperative toric IOL rotation of 5.7 degrees). [Fig. 2].

#### Binocular visual outcomes

At V0, mean binocular UDVA was 0.43 ± 0.21 LogMar, while mean CDVA was 0.31 ± 0.16 LogMar. Average UIVA at 80 cm and 60 cm were 0.53 ± 0.19 LogMar and 0.61 ± 0.18 respectively, while mean UNVA was 0.60 ± 0.20 LogMar.

All visual outcomes significantly improved compared after surgery. At V1, mean UDVA raised to 0.02 ± 0.03 LogMar (*p* < 0.0001), while CDVA was − 0.05 ± 0.07 LogMar (*p* < 0.0001). For intermediate vision, binocular UIVA at 80 cm and 60 cm improved to 0.02 ± 0.05 LogMar (*p* < 0.01) and 0.04 ± 0.07 LogMar (*p* < 0.01), respectively. Binocular CIVA was reportedly − 0.03 ± 0.06 both at 80 cm and 60 cm. At 40 cm, UNVA was 0.11 ± 0.04 LogMar (*p* < 0.01), while CNVA was − 0.04 ± 0.06.

### Refractive accuracy

**Figure 3:**
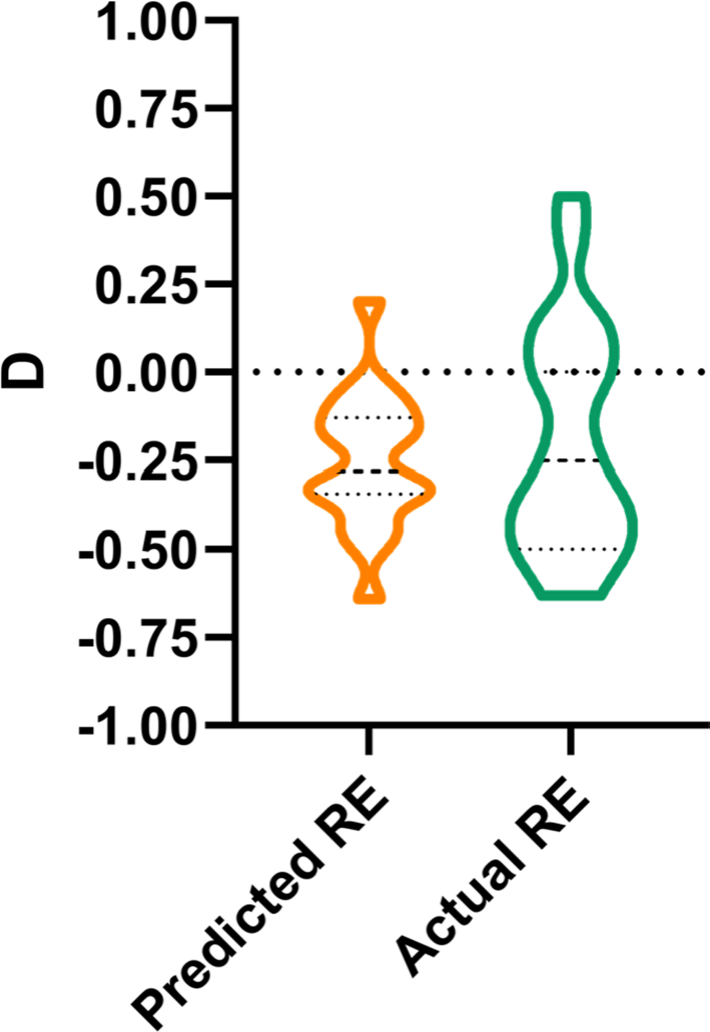
Violin plots showing the difference between biometric-predicted refractive error (RE) and measured refractive error measured by auto-refractometry. The mean difference between the predicted and measured RE, as spherical equivalent, was 0.09 D (*p* = 0.38).

At pre-operative biometric evaluation for entire cohort, mean predicted RE was − 0.25 ± 0.18 D (CI − 0.30 D, − 0.21 D). At 6-months follow-up, measured RE was − 0.20 ± 0.32 D (CI − 0.28 D, − 0.12 D), with a non- significant difference from predicted values (*p* = 0.38). The mean difference between the predicted and measured RE, as calculated by spherical equivalent, was 0.09 D. [Fig. 3].

In the toric-IOL group the PPRA, determined utilizing the Verion Image Guided System data, was 0.15 ± 0.08 at 96°, while the average RRA at V1 was 0.22 ± 0.32 at 92°. No statistical difference was found between PPRA and RRA (*p* = 0.06).

Similarly, in the non-toric IOL group the PPRA was 0.30 ± 0.09 at 103°, non-significantly different with the post-operative RRA at V1 (0.32 ± 0.09, *p* = 0.13).

#### Postoperative toric IOL rotation

On slit-lamp examination with dilated pupil, there was no apparent tilting of the IOL. In the setting of toric IOLs, when compared with the images acquired through the Verion Image Guided System at the end of surgery, we reported a mean postoperative toric IOL rotation of 2.1 ± 1.9 degrees (median 2.0 degrees; range 0–5,7 degrees) at V1. No IOL was misaligned by 10 degrees or more, while 1 eye had a IOL misalignment more than 5 degrees (5.7 degrees).

#### Additional results

Preoperative data showed that the mean anterior chamber depth was 3.22 ± 0.31 mm. Additionally, mean anterior chamber volume (mm^3^) and mean iridocorneal angle (degree°) were 136.5 ± 32.8 mm^3^ and 40.05 ± 7.88°, respectively. Photopic pupil size was between 3 and 4 mm for 33 (83%) of the patients, while 3 (7%) patients had a pupil < 3 mm and 4 (10%) had a pupil > 4 mm. The mean endothelial cell count before surgery was 2586 ± 329 cells/mm^2^, and the mean axial length was 24.57 (± 1.53) mm.

At V1, the mean anterior chamber depth was 4.13 ± 0.61, representing a 28.2% increase compared to V0. Postoperative measurements of mean anterior chamber volume (mm^3^) and mean iridocorneal angle (degree°), were 188.1 ± 25.79) mm^3^ and 52.95 (± 5.32)°, respectively indicating a relative increase of 37.8% and 32.2%, respectively, compared to baseline. Notably, there was no significant decrease (*p* = 0.11) in the mean endothelial cell count at V1 (2316 ± 372 cells/mm^2^).

### Spectacle independence

Twenty-one out of 40 patients (53%) reported total independence from their glasses at all distances (far, medium, and close). In the setting of computer work, 7 patients out of 40 (18%) admitted frequent glasses use. Four patients out of 40 (10%) patient sometimes used glasses for distant vision, in particular for night driving. Twelve patients out of 40 (30%) got used to near vision spectacle glasses for reading.

#### Patient satisfaction

**Figure 4:**
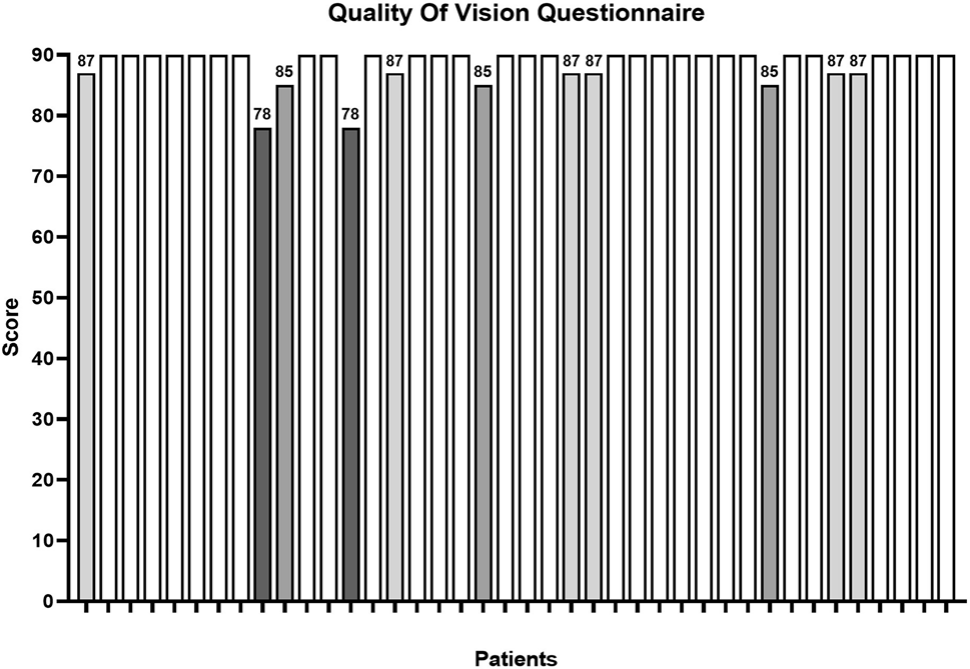
Histograms showing the results of Quality of Vision Questionnaire, out of 90 points, in all patients. Twenty-nine patients scored 90 out of 90 on the questionnaire, while lower values are for patients complaining for mild hazy or blurred vision (n = 3), halos (n = 3), glares (n = 2) or double images (n = 3).

Twenty-nine (72.5%) out of 40 patients scored 90 out of 90 on the questionnaire, referring no complaints in any situation. Moderate symptoms of distortion, hazy vision and blurred vision were reported in a minority of patients (n = 3, 7.5%). Few patients reported mild symptoms of disturbing light phenomena, such as halos (n = 3, 7.5%), glares (n = 2, 5%), or double images (n = 3, 7.5%). [Fig. 4] All of the patients reported that these symptoms appeared in the first month after surgery, but then tapered off during successive months. At the end of the study period, all patients had already become accustomed to these visual disturbances and none of them asked for lens replacement.

The result obtained by the static pupillometry analysis, as expected, influenced the Quality of Vision Score. Pupil size had indeed a positive correlation with the aforementioned score (r = 0.48, R^2^ = 0.23; = 0.002).

Overall, all of the patients reported satisfactory results with Alcon Vivity IOL and admitted than, if they had been asked again, they would have implanted the same lens.

## Discussion

The present study aimed to contribute valuable insights into the performance of the AcrySof IQ Vivity lens by evaluating visual and refractive outcomes. Additionally, in recognition of the comprehensive nature of prior research, the investigation aimed to provide additional insights into patients’ quality of life and satisfaction levels, particularly regarding reduced spectacle dependence.

Postoperative refractive errors and residual astigmatism have a negative impact on visual acuity and patient satisfaction, making them significant postoperative variables to be evaluated. The AcrySof IQ Vivity lens, although less effective than trifocal IOLs in correcting all visual distances, possess technological characteristics that make its management comparable to that of monofocal IOLs, as opposed to trifocal IOLs, which require additional considerations during surgical planning^17^.

Our findings regarding visual outcomes align with the current body of research^18^. In patients who received Toric IOL implantation, we reported a mildly superior refractive outcome both for UDVA and UIVA compared to Barber et al. results, while the residual RE is consistent with their investigation^19^ Furthermore, our research demonstrates that there were no statistically significant disparities between the group of patients who received toric and non-toric IOLs. As an additional point of interest, we discovered UNVA values that were marginally superior to those discovered by Jeon et al.^20^ UNVA were 0.18 ± 0.15 and 0.16 ± 0.06 LogMar for Toric and Non Toric, respectively vs. 0.22 ± 0.11) These results may be explained by the slightly smaller sample that we evaluated, as well as by the increased attention that was paid to preoperative and especially intraoperative approaches.

Moreover, a predictable postoperative refraction with accurate IOL power calculation (mean spherical equivalent difference between predicted and measured refractive error of 0.09D) was readily obtained by inserting tomography-measured corneal curvature indexes and optically measured axial length and anterior chamber depth into the Barrett II IOL formula. Furthermore, the use of Verion Image Guided System and intraoperatory monitoring of incision location, capsulorrhexis size, centering and toric IOL axis alignment was employed to standardize the procedure and maximize precision. Thanks to this technology, we were able to demonstrate that there were no statistically significant variations in refractive outcome between PPRA and RRA for both the toric and non-toric patient groups. These parameters alone were sufficient and provided a rapid and feasible preoperative planning approach in a busy real-world clinical setting.

According to the PRISQ questionnaire, more than half of patients (53%) reported complete independence from glasses at all distances (far, medium, and near) in their daily activities. Near vision correction with a spherical addition was the main cause of spectacle dependance (30%), which aligns with the expected performance of this model of IOL. However, spherical addition was mild with more than half of patients (60%) reaching maximum visual acuity with only + 1.00 D addition. This latest outcome is consistent with Arrigo’s research on the quality of life assessment^18^. Spectacles were rarely necessary for far vision in nighttime conditions, which is consistent with its reduced susceptibility to visual disturbances under low-light settings compared to other available multifocal IOL options^18^.

The impact on quality of life was significant, and a majority of patients (72.5%) reported no visual complaints and achieved the highest score of 90 on the Quality of Vision questionnaire. The visual disturbances that were reported the most frequently were haloes and glares. It was hypothesized that these visual disturbances would be positively connected with the results of pupillometry, which meant that participants with big pupils, greater than 4 mm, would have more disturbances. In light of this, it is very important to carry out a comprehensive evaluation of the size of the pupil before undergoing surgery.

No adverse events, including elevated intraocular pressure (> 21 mmHg) or hypotony (< 6.5 mmHg), endophthalmitis, postoperative macular edema, posterior capsule opacification (PCO) were observed and no additional medical or surgical treatments were administered. Patients exhibited a clear cornea at the end of the study follow-up with a decrease of 10.4% in endothelial cell count, which is consistent with literature findings^21,22^.

The main limitations to the present study include the small sample size and the monocentric nature of the study. Additionally, the lack of a comparison with referential normative data, the lack of defocus curves and the absence of the spatial frequencies analysis and of dynamic pupillometry represent limitations of this study. Moreover, the absence of a standardized questionnaires makes direct comparisons with other studies challenging^23–25^. Lastly, this investigation did not investigate aberrometry, which could have provided information about the impact of high-order aberrations on patient satisfaction and refractive outcomes.

In conclusion, the AcrySof IQ Vivity intraocular lens demonstrates safety and effectiveness in providing optimal refractive outcomes for distant and intermediate vision, leading to an improvement in patients’ quality of life with minimal to no visual disturbances. Additionally, over a half of patients achieved spectacle independence, further enhancing their overall satisfaction and a mild spherical addition was only required for optimal near vision. The IOL power calculations were accurately obtained through a streamlined and straightforward surgical planning process. With a potential decrease in cost over time, the AcrySof IQ Vivity may become a mainstay of future cataract surgeries. Further studies with standardized evaluation questionnaires will help to validate the findings and provide a more comprehensive understanding of its real life performance of in a broader patient population.

## Methods

The present study is a monocentric interventional study evaluating the preoperative and postoperative data of 40 patients (80 eyes) who underwent elective cataract surgery at the Ophthalmology Unit of Policlinico Agostino Gemelli IRCCS, Catholic University of the Sacred Heart—Rome, between February 2022 and September 2022.

The study received approval from the institution’s Ethics Committee and was conducted in accordance with the Declaration of Helsinki. Prior to enrollment, all patients provided written informed consent.

Inclusion criteria were the presence of a clinically significant cataract leading to a reduction of visual acuity. Additionally, the desire for spectacle independence among patients was taken into consideration as a criterion for recommending the AcrySof IQ Vivity lens. The benefits and potential risks associated with this lens were thoroughly explained during pre-operative assessments. Concomitant ocular conditions potentially affecting postoperative quality of vision such as severe dry eye disease, corneal opacity or ectasia, irregular or high astigmatism greater than 3 D, pathologic myopia, maculopathy, glaucoma, intraocular inflammation, previous keratorefractive surgery and a history of ocular surgery within 6 months prior to enrollment were considered to be exclusion criteria.

### Preoperative examinations

Prior to surgery (V0), all patients underwent a visual function assessment including LogMAR Early Treatment Diabetic Retinopathy Study chart (ETDRS) uncorrected and corrected distance visual acuity (UDVA, CDVA) at 6 m; uncorrected and corrected intermediate visual acuities (UIVA, CIVA) at 60 cm and 80 cm (ETDRS Chart, Precision Vision Inc., Woodstock, Illinois—100% contrast calibrated at 85 cd [cd]/m2); uncorrected and corrected near visual acuity (UNVA, CNVA) with a handheld chart at 40 cm under photopic condition and contrast sensitivity evaluation at 100 cm (Pelli–Robson Contrast Sensitivity Chart). All visual function tests were conducted for both monocular and binocular assessments. A comprehensive ophthalmic examination with Goldman applanation tonometry; slit-lamp biomicroscopy and fundus examination followed by spectral domain OCT examination of the macula (Spectralis HRA2 + OCT, Heidelberg Engineering, Heidelberg, Germany).

Surgical planning included axial length assessment through optical biometry (IOLMaster 500, Carl Zeiss Meditec, Germany), corneal keratometry through rotating Scheimpflug camera combined with Placido-disk tomography and static pupillometry (Sirius Topographer, CSO, Florence, Italy), and endothelial cell count (CEM-530; Nidek Co. Ltd, Gamagori, Japan). IOL power calculation was performed with the Barrett Universal II formula with emmetropia as refractive target^26^, and predicted refractive errors (RE) were collected.

Patients with preoperative astigmatism higher than 1.00D, were scheduled for AcrySof IQ Vivity Toric IOL implantation and power and orientation were calculated by elaborating the biometric measurements into the Alcon Toric IOL Calculator software (Alcon Laboratories Inc, Fort Worth, TX, USA). Surgically induced astigmatism (SIA) calculated by Verion Image Guided System was 0.14–0.16 D for all the patients.

### Surgical procedure

Surgical procedures were performed by two expert cataract surgeons (F.M.–D.L.) under topical anesthesia with oxybuprocaine drops. Following antisepsis with povidone-iodine solution all patients received a viscoelastic-assisted (Protectalon, VSY Biotechnology BV, Amsterdam, The Netherlands), coaxial, microincisional phacoemulsification surgery (Centurion Vision System, Alcon Laboratories Inc, Fort Worth, TX, USA) with a 2.2 mm main incision followed by the implantation of the AcrySof IQ Vivity IOL in the capsular bag. The main incision was superior and performed in the same position for all the patients. Patients received intraoperative antibiotic prophylaxes with the injection of 1 mg (0.1 ml) Cefuroxime (Aprokam, Laboratoires Théa, Clermont-Ferrand, France) in the anterior chamber. Intraoperative monitoring with Verion Image Guided System (Alcon, Fort Worth, TX) was performed to standardize the location of the main incision, the continuous curvilinear capsulorhexis diameter (5.5 mm) and centering on the pupillary axis and the final orientation of toric IOLs. Additionally, a digital color photograph was acquired through to be used as a reference to evaluate any postoperative complications, including toric IOL rotation.

Patients received standard postoperative care with topical steroids, NSAIDs and antibiotics and were closely monitored for adverse events at day 1, 15 and 30 days postoperative, at which time they were scheduled for surgery in the fellow eye. Successively,we distinguished eyes in two groups: Non-toric IOL implanted Group and Toric IOL implanted Group.

### Postoperative examinations

At 6 months after fellow eye surgery (V1), all the pre-operative examinations were repeated, i.e. ETDRS UDVA, CDVA, UIVA, CIVA, UNVA, CNVA, Pelli-Robson Contrast Sensitivity Chart, optical biometry, corneal keratometry, along with auto-refractometer evaluation (Nidek AR-1, Nidek Co., LTD, Maehama, Japan) to calculate measured post-operative refractive error.

#### Quality Of vision and spectacle independence questionnaires

In addition, two validated questionnaires were administered to evaluate the improvements in quality of life (QoL) following binocular implantation of the AcrySof IQ Vivity IOL. These questionnaires included the McAlinden’s Quality of Vision test and Morlock’s Patient-Reported Spectacle Independence Questionnaire (PRSIQ)^27,28^:

McAlinden’s Quality of Vision test was employed to collect patient-reported subjective visual discomfort, including symptoms such as blurred vision, glare, starbursts, haloes, foggy vision, distortion, difficulty concentrating, numerous images, and difficulty with depth perception. The test consists of 30 items, each scored between 0 and 3, with a total score range of 0–90 points. The questionnaire provides insights into the patients’ subjective experience: the higher the score, the greater the reported visual discomfort experienced by the patient.

The PRSIQ questionnaire consisted on the following questions:

- a)“Do you require spectacles or contact lenses?” with verbal response labels of “Yes” (1 point) and “No” (2 points).
- b)“How long have you worn spectacles or contact lenses?”.
- c)“How long were you able to function without spectacles or contact lenses?”.

For the “Wear” and “Function” items (b-c), the possible verbal responses and their respective scores were as follows: “All of the time” (scored as 1), “Most of the time” (scored as 2), “Some of the time” (scored as 3), “A little of the time” (scored as 4), “None of the time” (scored as 5). The scores obtained provide insights into the level of spectacle independence experienced by the patients.

Patients were asked to provide a verbal response of either “yes” or “no” to the question: “Considering the level of satisfaction with the quality of your vision without the use of spectacles, would you choose to undergo this intervention again in retrospect?”.

### Statistical analysis

Statistical analysis was conducted using GraphPad PRISM Software (Version 9.0; GraphPad, La Jolla, CA). The Shapiro–Wilk test was employed to assess the normality of the sample, with a p-value greater than 0.05 supporting the null hypothesis of normal distribution. For the comparison of continuous variables between baseline and postoperative data, Student’s paired t-test was utilized with a 95% confidence interval (CI), while the comparison between subgroups was performed with the Mann–Whitney U-test. Analysis of variance (ANOVA) was performed, and multiple comparison tests using matched pairs with Geisser-Greenhouse correction were conducted. Mean standard deviation (SD) was used to present quantitative data, and a significance level of *p* < 0.05 was employed to determine statistical significance.

### Ethical approval and consent to participate

This research was approved by the Catholic University of the Sacred Heart Ethical Committee in Rome, Italy. Written informed consent was obtained from participants to participate in the study.

### Institutional review

Board: Approval number 3961.

## Data Availability

Data is not publicly available due to ethical reasons. Further enquiries can be directed to the corresponding author, MMC, upon reasonable request.
